# Enablers and barriers for using reusable trocars: a qualitative study of surgeons’ and residents’ perspectives

**DOI:** 10.1007/s00464-026-12571-5

**Published:** 2026-01-26

**Authors:** Myrthe M. M. Eussen, Merijn de Kort, Carys Batcup, Merel L. Kimman, Brigitte A. B. Essers, Frenk van Harreveld, Nicole D. Bouvy, Monique Chambon

**Affiliations:** 1 Department of Surgery, Maastricht University Medical Center, P. Debyelaan 25, 6229 Maastricht, The Netherlands; 2 NUTRIM School of Nutrition and Translational Research in Metabolism, Maastricht University, Maastricht, The Netherlands; 3 Department of Social Psychology, University of Amsterdam (UvA), Amsterdam, The Netherlands; 4 Department of Clinical Epidemiology and Medical Technology Assessment (KEMTA), Maastricht University Medical Centre, Maastricht, The Netherlands; 5 Department of Surgery, Leiden University Medical Center, Leiden, The Netherlands

**Keywords:** Reusable trocars, Minimally invasive surgery, Implementation barriers, Sustainability, Behavioural determinants, Semi-structured interviews

## Abstract

**Background:**

Operating rooms (ORs) are among the most carbon-intensive areas of healthcare, with minimally invasive surgery (MIS) heavily reliant on disposable instruments. Trocars—essential for MIS and available in disposable and reusable forms—are major contributors to surgical emissions. Yet adoption of reusables remains limited. Effective measures to increase the use of reusables requires understanding the behavioral determinants of instrument choice. This study aimed to identify enablers and barriers to reusable trocar usage.

**Methods:**

We conducted a qualitative interview study guided by the Theoretical Domains Framework (TDF). Between September 2024 and May 2025, 20 surgeons and residents from seven Dutch hospitals participated in semi-structured interviews. Data were analyzed using a combination of deductive TDF coding, inductive coding and grouping into themes.

**Results:**

We identified 36 enablers and barriers grouped into nine themes: usability, workflow integration, training and skills, institutional systems, decision-making and ownership, culture and norms, safety, and perspectives on environmental and economic benefits. Most mapped to the TDF domains Beliefs about Consequences, Environmental Context and Resources, Skills, and Social/Professional Role and Identity. Usability concerns were manageable through surgical technique adaptations or design refinements. Workflow barriers diminished when reusables were integrated into trays and supported by clear sterilization protocols. Limited exposure during training reduced confidence, while demonstrations and senior role-modelling may facilitate uptake. Routine opening of disposables reinforced default single-use practices. Participants emphasized the need for environmental and economic data and noted the influence of procurement and management systems on the adoption.

**Conclusion:**

Many perceived enablers and barriers appear to stem more from environmental and contextual factors than from the devices themselves. Mapping our findings to the TDF highlights the domains that can guide implementation strategies. Coordinated, multi-level efforts may enable reusable trocars to become a practical and scalable intervention to reduce surgical emissions.

**Supplementary Information:**

The online version contains supplementary material available at 10.1007/s00464-026-12571-5.

Climate change is a global health threat [1]. Paradoxically, healthcare—intended to protect health—produces over 4.4% of global emissions and up to 10% in high-income countries [2–7]. This dual role of healthcare as both protector and polluter underscores the urgent need for sustainable practices, particularly in resource- and carbon-intensive settings such as operating rooms (ORs).

ORs are among the most emission-intensive areas in hospitals, responsible for 30% of hospital waste and a substantial share of healthcare’s carbon footprint [8–10]. Each surgical case can produce up to 814 kg carbon dioxide, varying by procedure and context [11–13]. Disposable medical devices and consumables are major contributors, making reduced reliance on disposables a key strategy to lower surgery’s environmental impact [14–20].

Minimally invasive surgery (MIS) has a higher carbon footprint than open surgery due to reliance on disposables such as trocars [12, 13, 21]. Widely available in both reusable and disposable forms, trocars are essential for MIS and rank among the top four contributors to its carbon footprint [22, 23]. Switching to reusable trocars could reduce emissions by up to 78%. [21, 24]

Despite broad support for sustainability in surgery, uptake of reusable instruments remains limited [22, 25–32]. Structural factors—such as costs, availability, and institutional policies—might play a role, while complex hospital systems may hinder further behavior change [33]. Education alone, though commonly used, has limited effectiveness in driving lasting change [20, 34–39]. Increasingly, social science and qualitative approaches are being recognized as vital to understanding aspects of surgical practice, including professionalism, education, and culture [40]. Applying these methods to sustainability can help identify determinants influencing reusables usage, enabling more effective interventions in the OR.

Implementation science frameworks, such as the Theoretical Domains Framework (TDF), provide a structure for exploring the determinants of behavior change [41–43]. The TDF synthesizes key psychological and contextual factors that influence professional behavior and is commonly used to guide interview design, data coding, and interpretation. This has been applied, for instance, by Davies et al. in studying reusable device adoption in the OR, though their focus was medical devices in general [44]. However, evidence shows that psychological influences on sustainable surgical behavior depend on the specificity of the behavior, meaning interventions are most effective when tailored to particular materials [45]. Building on this, our study uses the TDF to evaluate determinants influencing the use of reusable trocars. Using semi-structured interviews, we identified enablers and barriers to guide evidence-based interventions for increasing reusable trocar usage.

## Materials and methods

### Context and recruitment

This qualitative interview study was conducted within the CAREFREE project, a Dutch initiative for sustainable laparoscopy involving 11 hospitals. Seven hospitals were selected to ensure geographical and institutional diversity: two academic (Maastricht UMC + , UMC Groningen), three large general (≥ 500 beds; Isala, Rijnstate, Spaarne Gasthuis), and two small general (< 500 beds; Laurentius Hospital, Annadal Kliniek). Participants were surgeons and residents working with laparoscopic trocars. Inclusion criteria were: (1) employment at participating hospitals, (2) being a surgeon or resident, and (3) experience with trocars. Exclusion criteria were: (1) refusal to participate, or (2) lack of consent for audiorecording. Declines and non-responses were not systematically recorded.

Sequential sampling was used, combining convenience and snowball sampling. CAREFREE-affiliated surgeons received initial invitations and referred colleagues for snowball sampling. Predefined sampling targets were ≥ 2 surgeons and ≥ 2 residents per academic hospital, ≥ 1 surgeon and ≥ 1 resident per large hospital, and one surgeon per small hospital (where residents are not employed).

The sample size was guided by information power, appropriate for a focused aim, a relatively homogeneous participant group, despite variability across hospitals, and theory-driven interviews [43, 46–48]. Based on these factors and the use of interviews guided by the Theoretical Domain Framework (TDF), 16–20 interviews were anticipated to be sufficient.

Participants received the consent form via email ≥ 2 days in advance and provided verbal consent at the interview’s start, documented in the recording. The study was approved by the University of Amsterdam Ethics Review Board (FMG-UvA, FMG-10067) and reported according to the consolidated criteria for reporting qualitative research (COREQ) checklist. [49]

### Development of the interview guide

The semi-structured interview guide explored enablers and barriers to using reusable versus disposable trocars, guided by the Theoretical Domains Framework (TDF). The TDF includes 14 domains relevant to healthcare behavior (defined in Table 1), which guided the development of interview questions. Questions were also informed by prior research and interviews with experts in infection prevention, procurement, regulation, manufacturing, and sterilization, then mapped to TDF domains and refined with input from the CAREFREE team (surgeon, psychologists, qualitative experts). [22, 50] The interview guide was developed in Dutch and translated into English for reporting (Appendix A).

**Table 1:** Definitions of the 14 TDF domains as described by Atkins et al. (2017) [49]

TDF domain	Definition
Knowledge	An awareness of the existence of something
Skills	An ability or proficiency acquired through practice
Social/professional role and identity	A coherent set of behaviours and displayed personal qualities of an individual in a social or work setting
Beliefs about capabilities	Acceptance of the truth, reality or validity about an ability, talent or facility that a person can put to constructive use
Optimism	The confidence that things will happen for the best or that desired goals will be attained
Beliefs about consequences	Acceptance of the truth, reality, or validity about outcomes of a behaviour in a given situation
Reinforcement	Increasing the probability of a response by arranging a dependent relationship, or contingency, between the response and a given stimulus
Intentions	A conscious decision to perform a behaviour or a resolve to act in a certain way
Goals	Mental representations of outcomces or and states that an individual wants to achieve
Memory, attention and decision processes	The ability to retain information, focus selectively on aspects of the environment and choose between two or more alternatives
Environmental context and resources	Any circumstance of a person’s situation or environment that discourages or encourages the development of skills and abilities, independence, social competence and adaptive behaviour
Social influences	Those interpersonal processes that can cause individuals to change their thoughts, feelings, or behaviours
Emotion	A complex reaction pattern, involving experiential, behavioural, and physiological elements, by which the individual attempts to deal with a personally significant matter or event
Behavioural regulation	Anything aimed at managing or changing objectively observed or measured actions

### Interview conduct

All interviews were conducted by one researcher (ME), a medical doctor and PhD candidate, sometimes joined by a medical student. ME had no formal training in qualitative research techniques but was supervised by a multidisciplinary team. To minimize bias, ME introduced herself as independent researcher, though participants likely knew her affiliation. Her sustainability background may have influenced responses; to mitigate this, she emphasized that there were no ‘right’ answers and explicitly invited participants to discuss challenges, resistance, and reasons for continued disposable use. Neutral, open-ended questioning was used throughout.

Interviews were conducted via Microsoft Teams and scheduled to last about 45 min, with video and audio recording. Participant characteristics (department, role, experience, age) were collected during interviews. Field notes were not taken; all data were captured through recordings. No repeat interviews were conducted. Transcripts were generated with Amberscript, de-identified, and not reviewed by participants. Recordings were securely archived at the University of Amsterdam and deleted from Teams after upload.

### Data analysis

Transcripts were reviewed for accuracy and coded in Dutch using ATLAS.ti (v25.0.1). Data were analyzed through a three-phase framework analysis: (1) deductive coding within 14 TDF domains; (2) inductive coding to identify enablers and barriers; and (3) synthesis of enablers and barriers into overarching themes based on the primary level (e.g. individual, team, or organizational) and mechanism through which they influenced trocar use. Theme boundaries were iteratively discussed and refined within the research team to ensure conceptual clarity and internal coherence, while acknowledging interactions between themes. Themes were retained as distinct when they reflected different organizing principles, such as day-to-day workflow processes versus formal organizational structures or governance. Quotes were coded according to TDF domains; however, because some quotes related to multiple domains, data were presented by themes rather than by domains. Quotes relevant to illustrate the themes and corresponding TDF domains were translated into English (participant number; P = participant, S = surgeon, R = resident).

Two researchers (ME, MK) independently coded all transcripts using a predefined TDF codebook (Appendix B). After deductively and inductively coding the first three transcripts, they met to align interpretations; these transcripts were later recoded to check for drift. During analytic meetings, particular attention was paid to reflecting on how the interviewer’s background in surgical sustainability might influence interpretation, and alternative explanations were actively discussed between researchers. Broader reflections on surgical sustainability were retained if relevant. Coding discrepancies were rare and resolved through discussion; a third reviewer was unnecessary. Intercoder reliability was not calculated because of overlapping domains. [51]

## Results

Between September 2024 and May 2025, 20 semi-structured interviews were conducted with 12 surgeons and 8 residents, with a mean duration of 26 min and 52 s. Mean time in current role was 5 years, and laparoscopic experience 8 years. Although hospitals differed in size and organizational context, the identified enablers and barriers converged across settings. Thematic saturation—defined as the point at which no new enablers, barriers, or themes emerged—was reached after 18 interviews, with two additional interviews confirming saturation. Full participant characteristics are provided in Appendix C.

We identified 36 enablers and barriers, grouped into nine overarching themes. Most enablers and barriers were linked to the TDF domains Beliefs about Consequences, Environmental Context and Resources, Skills, and Social/Professional Role and Identity. Although all 14 TDF domains were represented, their prominence varied. The following sections describe each theme—developed from grouped enabler and barrier codes—and its associated TDF domain; a complete overview is provided in Table 2 and Appendix D.

**Table 2:** Key enablers and barriers for using reusable trocars, mapped to Theoretical Domains Framework (TDF) domains, with illustrative quotes

Enablers and barriers	Linked TDF domain	Example quotes
*Reusable trocars are perceived as less user-friendly*		
*Usability*		
Reduced usability in complex or technically demanding procedures	Beliefs about Consequences; Skills	“When it gets a bit more difficult, and you have more ports or need to switch frequently. It’s often more convenient to use one that stays in place better, and those are usually the disposables.” (P6-R) “If you look at the short procedures—like appendectomies and gallbladder surgery—I mainly do those with reusables.” (P1-S)
Peritoneal laxity increases technical difficulty when inserting reusable trocars	Skills	“To be honest, the suprapubic five-millimeter reusable trocar is a bit tricky. You need extra skill to insert it properly into the abdominal wall so the peritoneum doesn’t endlessly bunch up around the trocar. In that case, a disposable is easier.” (P2-S)
Dissatisfaction with reusable trocar usability reinforces disposable use	Reinforcement	“There’s also dissatisfaction with how intuitive the reusable trocars are, which makes people dig in their heels and default to using disposables.” (P2-S)
Compatibility concerns with surgical instruments	Beliefs about Consequences	“In our tray, there are standard instruments that could be used for the surgery, but they don’t fit through those reusable trocars. So then you still have to unpack a disposable trocar to get, for example, the clip applier through it, or you have to unpack a disposable clip applier. You want trocars that fit all instruments, or you need a tray that is coordinated—that would make switching much easier.” (P1-S)
*Design and ergonomics*		
Weight and design reduces ergonomics	Beliefs about Consequences	“I think those reusable trocars really hang heavily on the abdomen. They’re quite heavy because they’re made of some kind of metal. So all in all, I just find the way they feel in the hand, the placement, and the way they hang on the belly during the procedure less comfortable. All of that together makes them less easy to use.” (P8-R) “I have the impression that equipment made to be reused is well-made and of high quality. Because if it’s not strong enough, you can’t reuse it properly. So I think the material quality is good.” (P19-R)
Complexity to handle due to added procedural steps	Beliefs about Consequences	“Reusable trocars are a bit more cumbersome. You have to use a valve, for example, to switch between five and ten millimeters.” (P12-S) “If you have a reusable trocar that’s just as good—or nearly equivalent—in terms of ease of use, and the price isn’t much higher than a disposable, then the barrier to switching is very small.” (P15-S)
Fixation and anchoring concerns	Beliefs about Consequences	“Yes, there’s a little balloon attached to the disposables—inside the abdomen there’s a balloon, and on the outside, some kind of plastic, rubber, or silicone slider that helps fixate it in the abdominal wall. That fixation is useful.” (P2-S) “The trocars we use now have this useless little balloon that only requires you to make a slightly larger incision for the patient.” (P3-S)
*Performance*		
Air leakage due to design or reducer use	Beliefs about Consequences	“I think the larger reusable trocars are the real pain points because you need a reducer during surgery. If you’re using a smaller instrument, you have to grab a reducer—if you don’t, it leaks like crazy. That extra step often goes wrong, especially when we want to switch from a large to a small instrument in one go." (P3-S) “You get more air leakage with the five-millimeter ones if the incision is too large when screwing it in, and with the ten-millimeter ones if the open introduction is too wide. This happens more often with reusables. With disposables, you can use the Veress needle, and the incision in the fascia matches the size of the trocar exactly.” (P1-S) “If you get a lot of leakage and it makes the surgery harder to perform, that will be a major obstacle to implementation.” (P7-S) “You could argue that it takes a bit longer to insert the reusables because with the one I’ve used, you really have to screw it in. And the Hasson type sometimes leaks, the larger port sometimes leaks CO₂ because it’s not sealed properly.” (P10-R)
*Mixed perceptions of workflow efficiency with reusable trocars*		
Surgical culture prioritizes speed	Social/Professional Role and Identity	“I think a lot of surgeons prioritize ease of use and speed. There’s always some pressure on operating time and similar concerns.” (P3-S) “If in the end it takes five extra minutes to be more environmentally conscious and things like that, then I’d still do it.” (P10-R)
Additional handling steps during post-operative processing	Beliefs about Consequences	“Cleaning up a disposable trocar is a single movement, just throw it in the bin. With the reusables, they might have to do something with them in the OR, maybe some rough cleaning for visible debris. […] OR nurses also have to repack the net that contains all the instruments and trocars, nothing can be missing.” (P2-S) “I don’t know whether it’s more or less work for the scrub nurse. I don’t think it makes much of a difference.” (P10-R)
Tracking risks of reusable instruments	Environmental Context and Resources	“Especially during cleanup, you need to be careful not to roll up the drapes with a trocar still inside. That’s where I see the biggest issues, sometimes a trocar might accidentally get thrown away. We really need to be careful about counting them.” (P5-S)
Increased workload for CSSD	Beliefs about Consequences	“That will undoubtedly be more work. They need to be cleaned, sent to the CSSD, and packed in a sterile way. So that’s extra work either way.” (P6-R) “In our hospital, the reusable trocars are already in the tray with all the reusable instruments, so they just get sterilized along with everything else. It’s not an additional sterilization process.” (P15-S)
*Limited exposure and training reduce confidence in using reusable trocars*		
Limited exposure to reusable trocars during training	Environmental Context and Resources; Skills	“So I think residents go for disposable ones, especially in the beginning when they don’t do much laparoscopic surgery. They often don’t even know about reusables or don’t use them, because in many other hospitals reusable trocars aren’t used at all.” (P8-R) “That depends on how often you use the trocars. They do require some skill, not extreme, and you can teach it to any monkey. But you have to be willing to do it, and if it’s handed to you right away, then you need to have done it a few times first. Same goes for the disposables.” (P2-S)
Declining training in open technique limits confidence with reusable trocars	Skills	“Preferably, the first trocar is introduced using the open technique, which also requires a certain skill. I always do it that way, but you can see that fewer and fewer surgeons do it now, because they think using a Veress needle is also safe. So if, for example, your supervisor suddenly asks you to use a reusable trocar for the open introduction, that’s quite difficult.” (P19-R) “And if you ask someone to insert a reusable, then you’re forcing them to do an open introduction, which is also a good technical skill that every surgeon should have, in my opinion. Open surgical introduction simply a technique you should master.” (P1-S)
Initial unfamiliarity with reusable trocars creates risk of error	Skills	“The downside of a reusable is that if you’ve never worked with it before and it’s just handed to you by the OR nurse—who may also have never used it—then you can get into trouble. So it really needs a short instruction where you’ve actually held it in your hands at least once.” (P1-S) “Suppose we make the switch, then the company supplying the trocars should come and give an info session or training on how they work, so we can all hold it in our hands once… and after that, very practically, just start using it in the OR.” (P8-R)
*Reusable trocars dependent on reliable logistics and availability*		
Disposable trocars are perceived as offering more logistical independence	Environmental Context and Resources; Beliefs about Consequences	“That’s the advantage of disposables, you can always open a cabinet and there the disposables are. You need less infrastructure for cleaning, transport, and instrument trays that could become unsterile. I think that’s the biggest hurdle.” (P18-S) “You don’t have to deal with supply issues from external companies because you’ve got your own sets that you can sterilize. So as long as your facilities are in order, it should be fine.” (P9-S)
Delays in repair or replacement of reusable trocars due to supplier dependency	Environmental Context and Resources	“If a reusable trocar breaks and you’re dependent on certain companies, you can end up waiting endlessly for service before getting your equipment back.” (P17-S)
*Decision-making and ownership dynamics hinders adoption of reusable trocars*		
*Surgeons excluded from decisions*		
Lack of clinician involvement in procurement decisions	Social Influences	“Sometimes we’re simply presented with the fact that certain agreements—about sutures or instruments, for example—have already been made, without all surgeons being informed or involved in testing them beforehand.” (P1-S) “There are decisions you don’t need to be involved in. As a surgeon, you don’t need to be included in everything, but if something impacts your practice—like trocars -, it’s often nice to be informed.” (P13-S)
Residents excluded from decision-making processes	Social/Professional Role and Identity	“I think that decision should lie with the person using the instruments: the surgeon. I don’t think residents need to be involved, since they rotate through many hospitals and learn to use different tools, which is very educational. In my view, the surgeon, gynecologist, urologist, or whoever’s performing the procedure should be involved in making that decision.” (P16-S)
*CSSD and procurement challenges*		
Resistance from CSSD can obstruct adoption of reusable trocars	Social Influences	“Definitely the CSSD, they’re the biggest stakeholder. I’ve had quite a battle there. We tried—well, I tried—to introduce reusable trocars two years ago, but in the end it was blocked because they didn’t pass a certain test or inspection. So yes, they’re a key player.” (P17-S)
Procurement decisions are dominated by cost, limiting clinician and environmental input	Social/Professional Role and Identity	“What I often see in this system is that another department ultimately says, ‘This is the cheapest option, so that’s what you’ll use.’ They follow the board’s direction: either minimize costs, or—if the goal is to present ourselves as a truly green hospital—accept some additional costs and go with reusables.” (P3-S) “If the surgeons say, ‘We want this,’ then it will have to be implemented. They’re the ones who can ultimately make it happen.” (P7-S)
*Environmental responsibility seen as organizational*		
Disconnect between clinical and institutional sustainability responsibilities	Social/Professional Role and Identity	“Of course, there’s the environmental impact and the footprint we leave behind. But as a surgeon, I don’t directly notice that—only at the organizational level.” (P17-S)
Hospitals underutilize procurement to promote sustainable design	Social/Professional Role and Identity	“I think hospitals have a real responsibility to tell manufacturers they need to make their products reusable. And if they don’t, just don’t buy from them. That’s what it takes to drive change.” (P19-R)
*Mandates resisted without evidence*		
Resistance to mandated implementation without evidence	Social Influences	“I don’t think mandatory use really works. You need to convince people with data and arguments. If that doesn’t work, then your case isn’t strong, so even if you try to mandate it, it likely won’t work.” (P18-S)
Overreliance on voluntary individual behavior	Social/Professional Role and Identity	“When it comes to decisions like these, if you leave everything up to the users, nothing will ever change.” (P5-S) “At a certain point, I think decisions need to be made from the top, because otherwise people won’t take action themselves. I’m completely fine with that.” (P18-S)
Lack of formal mandates to ensure consistent adoption	Environmental Context and Resources; Social Influences	“If the organization fully supports it and sets the standard as ‘we use reusables now,’ then everyone will have to switch.” (P7-S)
*Resistance to change shaped by surgical culture and norms*		
Reluctance to change due to discomfort	Emotion	“There’s always a group of people who aren’t fond of change, and they’ll look for reasons not to change. Whether those reasons are valid, I don’t know…” (P19-R) “It might also help to give some instructions on how to use them. I think the ‘why’ behind it is really important. If you explain that clearly, I believe many people would be open to it and that would make the transition a lot easier.” (P14-S)
Healthcare norms and surgical culture discourage change	Social/Professional Role and Identity	“I know colleagues who use more disposables. One of them, a fellow, really uses a lot and I always call him out on it. […] He says he’s not used to them and doesn’t really care about the whole Green OR concept.” (P16-S) “I think there’s increasing social pressure, since sustainability is a broader societal issue. More and more people are affected by it and want to take action, so yes—I do think the pressure around sustainability is growing.” (P13-S)
Institutional workflows and routines reinforce disposable use	Social Influences; Environmental Context and Resources; Behavioral Regulation; Goals	“It depends on their preference, because often, by the time you start the operation, the OR nurse has already opened a set with all the disposable trocars i- since those are still the default, even when reusables are available.” (P7-S) “People often reach for disposable plastic trocars without much thought, I get the sense it’s not really considered; they just use whatever they’re given.” (P2-S) “If you really want to implement it, I think you should just eliminate the disposable trocars altogether. That way, people won’t have a choice anymore and I think that would make a big difference.” (P8-R)
*Patient safety dominates instrument choice*		
*Safety considerations drive instrument choice*		
Limited visibility during optical introduction	Beliefs about Consequences	“Because we do an optical introduction, the tip is transparent, so you can actually see whether you are entering the bowel. And of course, if you are in the bowel, you realize it's too late but at least you’ve seen that you’re in it.” (P15-S) “For the non-open introduction—introduction under vision—and the use of the Veress needle, a disposable is often used that allows you to see the organs during insertion. That is a surrogate—I use that word deliberately—a surrogate safety that many people feel comfortable with.” (P2-S)
Perceived safety risks related to energy use	Beliefs about Consequences	“There’s a theoretical risk of current leakage because they’re metal, whereas plastic trocars don’t conduct. So when using monopolar energy, current could theoretically conduct through the metal trocar and cause off-screen damage. For example, thermal injury to the bowel if it’s nearby. That’s a theoretical drawback of the reusable.” (P6-R) “I think that issue is long resolved, but some people still wonder: is it safe to have metal that could theoretically be in contact with an organ?” (P18-S)
Preference for disposables in specific patients	Beliefs about Consequences	“For patients with a higher BMI, there’s a kind of extra part of the trocar that you can cut off to create more working space in specific patients. And for very young children—up to about six years old—disposables can sometimes work better. But those are rare exceptions.” (P7-S)
Sustainability deprioritized when safety or ease is at stake	Beliefs about Consequences; Social/Professional Role and Identity	“If choosing a particular trocar makes the procedure more difficult for yourself or less safe for the patient, then I believe patient safety and ease of use should always outweigh the small amount of additional waste it may produce.” (P15-S) “I think it’s important because this is a small, simple choice surgeons can make to reduce the environmental impact of surgery.” (P16-S)
*Patients focus on safety, not instrument choice*		
Focus on patient safety and surgeon confidence	Memory, Attention and Decision Processes	“During surgery, it doesn’t matter to a patient what instruments are used. They just want a safe and successful operation. They want a surgeon who feels comfortable and confident at the table—that’s really what matters to them.” (P3-S) “I don’t think patients are told much about the instruments. It probably doesn’t matter much to them either, but I do think they’d appreciate it if we make choices that are better for the environment.” (P10-R)
*Uncertainty about the environmental benefits of reusables*		
Concerns about environmental trade-offs of sterilization	Beliefs about Consequences	“If you have to sterilize it, how much microplastic ends up in the surface water? And how many harmful substances are released into the environment? […] Because if we’re going back to using sterilization chemicals that are highly toxic or release microplastics into wastewater, we might still be causing harm.” (P18-S)
*Perceived cost savings but upfront investments remain a challenge*		
Reusables cost more	Beliefs about Consequences	“In terms of cost, there’s obviously an initial investment required, and I can see how that might become an issue. In the long run, it’s undoubtedly cheaper but I can also imagine that making that upfront purchase could be a problem for some hospitals.” (P5-S) “At our hospital, reusable trocars have actually been standard for years. That started long before sustainability became a concern—there was a financial motivation behind it." (P9-S)

*CSSD* Central Sterile Services Department, *P* Participant, *S* Surgeon, *R* Resident

### Reusable trocars are perceived as less user-friendly

This theme encompasses enablers and barriers mapped to the TDF domains Beliefs about Consequences, Skills and Reinforcement.

#### Usability

Reduced usability in technically demanding procedures was mentioned. In surgeries with multiple ports or frequent instrument switching, disposables were preferred: *“When it gets a bit more difficult […] it’s often more convenient to use one that stays in place better, and those are usually the disposables.”* (P6-R). Insertion difficulties, especially with abdominal wall laxity, were also noted.

Participants expressed general dissatisfaction with the usability of reusables: *“There’s also dissatisfaction with how intuitive the reusable trocars are, which makes people dig in their heels and default to using disposables.”* (P2-S).Opinions on screw-type reusable trocars were mixed, seen as time-consuming or more prone to leakage, while others noted that with careful insertion and small incisions, they fit securely.

Instrument compatibility also influenced usability. Mismatches between reusable trocras and other laparoscopic instruments occasionally forced a return to disposables:*“You want trocars that fit all instruments, or you need a tray that is coordinated.”* (P1-S).

#### Design and ergonomics

Design-related challenges included heavier weight and metallic construction of reusables, which were perceived to reduce ergonomics: *“[…] reusable trocars really hang heavily on the abdomen. […] All of that together makes them less easy to use.”* (P8-R). Yet, others viewed this weight as a marker of quality and durability, reflecting differences in personal preference, or instrument expectations. Some emphasized that if reusables offered ease of use comparable to disposables, switching would present little barrier:*“If you have a reusable trocar that’s just as good […] then the barrier to switching is very small.”* (P15-S).

Fixation—how securely a trocar remains in the abdominal wall—was another concern. Some valued the balloon in disposable trocars for stability, while others dismissed it as unnecessary and requiring a larger incision:*“[…] useless little balloon that only requires you to make a slightly larger incision […]”* (P3-S).

#### Performance

Air leakage was one of the most frequently discussed performance issues, particularly with reusables. Problems often arose when switching between instrument sizes using reducers: *“If you’re using a smaller instrument, you have to grab a reducer—if you don’t, it leaks like crazy.”* (P3-S). Leakage was also attributed to poor fit, incision size, or valve maintenance. Improperly cleaned valves were said to worsen leakage and complicate surgery: *“If you get a lot of leakage and it makes the surgery harder to perform, that will be a major obstacle to implementation.”* (P7-S).

### Mixed perceptions of workflow efficiency with reusable trocars

This theme encompasses enablers and barriers mapped to the TDF domains Social/Professional Role and Identity, Beliefs about Consequences, and Environmental Context and Resources.

Efficiency demands and time pressure led many to prefer disposables: *“[…] lot of surgeons prioritize ease of use and speed. There’s always some pressure on operating time.”* (P3-S). Yet some were willing to accept minor delays if this contributed to environmental benefits:*“If in the end it takes five extra minutes to be more environmentally conscious, then I’d still do it”* (P10-R).

Extra intraoperative steps specific to reusables—such as switching sizes via valves—were seen as slowing procedures. Additional postoperative handling by OR nurses, such as repacking nets with instruments and trocars, was likewise described as disruptive: “*OR nurses might have to […] repack the net that contains all the instruments and trocars.”* (P2-S). Others, however, considered this negligible compared to discarding disposables.

Postoperative tracking of reusables was another concern, as they were considered more prone to accidental disposal during clean-up:*“Sometimes a reusable trocar might accidentally be thrown away. We really need to be careful about counting them.”* (P5-S). This risk was said to require strict workflows and consistent counting protocols.

Central sterile services department (CSSD) workload was frequently mentioned. Surgeons described delays in instrument delivery due to staff shortages, with reusables adding to this burden: *“That will undoubtedly be more work. They need to be cleaned, sent to the CSSD, and packed in a sterile way.”* (P6-R). Yet in hospitals where reusable trocars were already integrated into trays, CSSD workload was not considered problematic:*“In our hospital, the reusable trocars are already in the tray […], so they just get sterilized along with everything else.”* (P15-S).

### Limited exposure and training reduce confidence in using reusable trocars

This theme encompasses enablers and barriers mapped to the TDF domains Environmental Context and Resources and Skills.

Residents defaulted to disposables because reusables were rarely available in training settings, limiting both experience and awareness: *“They often don’t even know about reusables or don’t use them, because in many other hospitals reusable trocars aren’t used at all.”* (P8-R).

Insufficient training in open insertion was also mentioned. As open approaches are less frequently taught, switching to reusables requiring this method was challenging: “*[…].if your supervisor suddenly asks you to use a reusable trocar for the open introduction, that’s quite difficult.”* (P19-R). Others framed this decline as a missed opportunity to maintain essential skills:*“Open surgical introduction, a simple technique you should master.”* (P1-S).

Unfamiliarity was perceived as risky without prior instruction:*“If you’ve never worked with it before and it’s just handed to you […] then you can get into trouble.”* (P1-S). Brief hands-on training by suppliers was suggested to build confidence: *“The company supplying the trocars should come and give an info session or training on how they work, so we can all hold it in our hands once … and after that, very practically, just start using it in the OR.”* (P8-R).

### Reusable trocars dependent on reliable logistics and availability

This theme encompasses enablers and barriers mapped to the TDF domains Environmental Context and Resources and Beliefs about Consequences.

Disposables were valued for reliability and immediate availability, independent of sterilization:*“You can always open a cabinet and there the disposables are.”* (P18-S). Reusables were considered advantageous when sterilization logistics were reliable, highlighting reduced supply problems compared to disposables: “*You don’t have to deal with supply issues […] because you’ve got your own sets that you can sterilize.”* (P9-S). Still, repair and replacement delays, vendor dependence, CSSD workload, and staff shortages risked reducing availability and letting malfunctioning instruments circulate, disrupting workflow.

### Decision-making and ownership dynamics hinders adoption of reusable trocars

This theme encompasses enablers and barriers mapped to the TDF domains Social Influences, Social/Professional Role and Identity and Environmental Context and Resources.

#### Surgeons excluded from decisions

Surgeons noted being excluded from procurement, with trocar contracts sometimes decided without prior testing: *“Sometimes we’re simply presented with the fact that certain decisions […] have already been made, without all surgeons being informed or involved in testing them beforehand.”* (P1-S). While full consensus was not deemed necessary, consultation was valued when choices affected surgical practice: *“If something impacts your practice—like trocars—it’s often nice to be informed.”* (P13-S).

Surgeons felt residents need not to be involved in procurement discussions due to their rotational training:*“I don’t think residents need to be involved, since they rotate through many hospitals […]”* (P16-S). Residents themselves did not emphasize procurement involvement.

#### CSSD and procurement challenges

CSSD and procurement systems were sometimes viewed as blocking reusables, either through testing requirements or cost-driven decisions. One participant described a failed attempt due to CSSD resistance: *“[…] the CSSD, they’re the biggest stakeholder. I’ve had quite a battle there.”* (P17-S). Others cited procurement focusing mainly on price: *“Another department ultimately says, ‘This is the cheapest option, so that’s what you’ll use.”* (P3-S). Still, clinician leadership was seen as an enabler to convince CSSD and procurement of reusables’ necessity.

#### Environmental responsibility seen as organizational

Many clinicians felt disconnected from their hospital’s sustainability agenda, viewing environmental impact as an organizational rather than clinical responsibility: *“There’s the environmental impact and footprint we leave behind. But as a surgeon, I don’t directly notice that—only at the organizational level.”* (P17-S). Surgeons emphasized that hospitals must reduce environmental harm, including through procurement decisions: *“Hospitals have a real responsibility to tell manufacturers they need to make their products reusable. And if they don’t, just don’t buy from them.”* (P19-R).

#### Mandates resisted without evidence

Resistance to mandated use of reusable trocars was expressed, especially without supporting evidence. Clinical data and persuasive arguments were considered more effective than enforcement: *“You need to convince people with data and arguments […] even if you try to mandate it, it likely won’t work.”* (P18-S). At the same time, voluntary action by individual surgeons was seen as insufficient: *“If you leave everything up to the users, nothing will ever change.”* (P5-S). Institutional mandates were viewed as essential by some participants, provided they were grounded in patient safety or quality standards: “*If the organization fully supports it and sets the standard […] then everyone will have to switch.”* (P7-S).

### Resistance to change shaped by surgical culture and norms

This theme encompasses enablers and barriers mapped to the TDF domains Emotion, and Social/Professional Role and Identity, Social Influences, Environmental Context and Resources, Behavioural Regulation and Goals.

Discomfort with changing routines was often mentioned. Participants noted reluctance among colleagues to adopt new systems: *“There’s always a group of people who aren’t fond of change, and they’ll look for reasons not to change.”* (P19-R). Others stressed that effective communication could reduce this resistance: *“[…] the ‘why’ behind it is really important. If you explain that clearly, I believe many people would be open to it.”* (P14-S).

Healthcare norms also shaped resistance. Some resisted reusables out of personal preference or skepticism: *“He says he’s not used to them and doesn’t really care about the Green OR concept.”* (P16-S). Others, however, observed *“increasing social pressure”* (P13-S) toward greener practices.

Disposable trocar use was often described as automatic, with little attention to sustainability: *“People often reach for disposable plastic trocars without much thought.”* (P2-S). OR nurse routines further reinforced disposables as default: *“The OR nurse has already opened a set with the disposable trocars—since those are still the default.”* (P7-S). In contrast, switching to reusables was seen by some as fostering awareness of surgical waste: *“It also creates a kind of awareness that you’re trying to minimize disposable use because you’re consciously thinking about it.”* (P11-R). Some suggested eliminating disposables altogether to shift these ingrained practices: *“If you really want to implement it, […] you should just eliminate the disposable trocars altogether.”* (P8-R).

### Patient safety dominates instrument choice

This theme encompasses enablers and barriers mapped to the TDF domains Beliefs about Consequences, Social/Professional Role and Identity, Memory, Attention and Decision Processes.

#### Safety considerations drive instrument choice

Limited visibility during insertion, particularly among surgeons using visual introduction, was a concern. Transparent disposable trocars were seen as offering insertion security, though not universally: *“That is a surrogate safety […] that many people feel comfortable with.”* (P2-S). Here, the surgeon referred to the visibility providing a sense of safety, rather than proven safety benefits.

Perceived risks during energy use also contributed to hesitation, particularly with metal reusable trocars. Some raised the theoretical risk of stray current in electrosurgery: *“There’s a theoretical risk of current leakage […] and could cause off-screen damage […]”* (P6-R). Others considered this outdated: *“I think that issue is long resolved.”* (P18-S). These differing views appeared to reflect varying levels of knowledge and familiarity with current device safety standards.

Disposables were valued in specific contexts, such as pediatric or high-BMI patients. One participant referred to a feature of disposable trocars that allowed adjustments (“a purple ring”), which created additional working space: *“There’s a kind of extra part of the trocar […] that you can cut off to create more working space.”* (P7-S).

Beyond specific safety concerns, many participants viewed sustainability as secondary to safety and ease of use:*“If choosing a particular trocar makes the procedure more difficult for yourself or less safe for the patient, then I believe patient safety and ease of use should always outweigh the small amount of additional waste it may produce.”* (P15-S). Still, some emphasized that small choices, like trocar use, form part of surgeons’ professional responsibility toward sustainability*: “This is a small, simple choice surgeons can make to reduce the environmental impact of surgery.”* (P16-S).

#### Patients focus on safety, not instrument choice

Most felt patients cared mainly about safety and outcomes, rather than instrument usage: *“It doesn’t matter to a patient what instruments are used. They just want a safe and successful operation. […] That’s really what matters to them.”* (P3-S). Patients were generally seen as unaware of instrument choice. Some suggested rising patient awareness of environmental issues might support reusables, even if such choices remain invisible for them.

### Uncertainty about the environmental benefits of reusables

This theme encompasses enablers and barriers mapped to the TDF domains Beliefs about Consequences.

Some questioned whether reusables were inherently more sustainable, citing sterilization-related harms:*“If you have to sterilize it, how much microplastic ends up in the surface water? […] We might still be causing harm.”* (P18-S).

### Perceived cost savings but upfront investments remain a challenge

This theme encompasses enablers and barriers mapped to the TDF domains Beliefs about Consequences.

Most participants believed that reusables could lead to cost savings over time, but emphasized that high upfront investments could limit adoption at hospital level: *“There’s obviously an initial investment required, […] making that upfront purchase could be a problem for some hospitals.”* (P5-S). Financial considerations were also seen as an enabler. Some noted that reusables had already been introduced in their hospitals before sustainability was prioritized, suggesting that cost savings had motivated the change: *“At our hospital, reusable trocars have actually been standard for years. That started long before sustainability became a concern—there was a financial motivation behind it.”* (P9-S).

## Discussion

This study applied the Theoretical Domains Framework (TDF) to explore factors influencing adoption of reusable trocars. Disposable trocars substantially contribute to emissions from MIS, yet reusable alternatives remain underused despite broad availability [21–24]. Using the TDF, we identified enablers and barriers related to usability (including design, ergonomics, and performance), workflow integration, training and skills, institutional systems, decision-making and ownership, culture and norms, safety, and perspectives on environmental and economic sustainability.

Usability concerns such as air leakage, insertion difficulty, or limited visibility were noted but often manageable with technique or design improvements. Features like heavier weight were seen as barriers by some and as signs of quality by others, indicating that concerns reflected individual preferences and professional routines more than device flaws. These findings align with research showing that innovations misaligned with clinical norms face resistance, while medical device development often undervalues broad end-user input, underscoring the need to integrate diverse perspectives early in the design phase. [52, 53]

Adoption was shaped not only by individual preferences but also by institutional integration. While reusables are often perceived to overburden workflows and sterilization services, our findings nuance this: in hospitals where reusable trocars were included in trays, disruption was minimal and workflow concerns diminished with experience, a benefit also highlighted during supply shortages such as COVID-19 [44]. Since sustainability efforts fail without operational alignment, integration strategies must be tailored to local contexts, including Central Sterile Services Department (CSSD) capacity, staffing, and tray logistics. [54–56]

Institutional support and procurement policy were viewed as decisive levers for sustainable change. Hospitals can shape markets by phasing out disposables and setting clear sustainability criteria, aligning with systems thinking that recognizes procurement as a strategic tool for decarbonization [57–59]. Frameworks such as the 10R circular economy model—which ranks 10 strategies from high-impact options like refusing and reducing to lower-impact options like recycling and recovering—can help prioritize actions and avoid superficial “greenwashing” [60, 61]. Clinicians welcomed institutional backing but stressed that procurement choices must rest on robust clinical, financial, and environmental data. Views on mandating reusables varied: some surgeons expressed openness to adopting new practices, while others described frustration with colleagues who were hesitant to change. Reusables—long used early in laparoscopy—challenge the assumption that disposables equal progress. Participants emphasized that sustainable change requires leadership, clear ownership, and organizational commitment [62–64]. Yet, as shown in the qualitative study by Davies et al. on reusable device adoption in the operating room (OR)—and as our findings confirm—responsibility for reusable instruments is often poorly defined, leaving unanswered who should decide on instrument choice and how to balance performance, cost, and environmental impact. [44]

Limited exposure and training, particularly during residency when disposables dominated, reduced confidence in reusables. Declining use of open insertion techniques—required for some reusables due to a lack of an insufflatable balloon—limited essential skill retention. Participants emphasized hands-on training, supplier demonstrations, and senior surgeon support to build confidence. Early-career exposure is crucial for shaping durable, climate-conscious surgical habits. [65]

Surgical culture and norms also influenced adoption. Some surgeons resisted reusables due to established routines, personal preference, or skepticism toward sustainability, while others described increasing social pressure—an enabler—to adopt greener practices. OR nurses’ routines further reinforced disposables as the default in many cases, disposable sets were opened in advance as part of standard preparation. Several participants suggested eliminating disposable options altogether to disrupt ingrained routines. Clear communication about sustainability goals and credible evidence on environmental and financial impacts are essential to overcome resistance and beliefs that reusables are costly or less eco-friendly. [44]

While patient safety and ease of use remained priorities, reusables were described as a low-effort, high-impact step toward sustainable practice, a finding echoed by Davies et al. [44] Reusables were also seen as catalysts for broader awareness of surgical waste. Some participants suggested that rising patient concern about sustainability might indirectly support adoption, though patients typically prioritize health outcomes [66]. These findings raise ethical questions about whether environmental impacts should be included in decision-making, particularly as disposables contribute to climate change. Emerging “green bioethics” highlights balancing autonomy with responsibility toward planetary health. [67, 68]

Applying the TDF helped move beyond simply describing enablers and barriers toward identifying where innovation and implementation should focus [41–43]. The most frequently coded domains—Beliefs about Consequences, Environmental Context and Resources, Skills, and Social/Professional Role and Identity—show that trocar use is shaped more by workflow constraints, skill confidence, and surgical culture than by the devices themselves. Our findings also align with earlier work indicating that Social/Professional Role and Identity and Environmental Context and Resources are key determinants of reusable device adoption [36]. Together, these patterns offer guidance for de-implementing disposables and introducing reusables, including addressing usability and leakage concerns, ensuring reliable logistics, and improving training and exposure. Shifting entrenched norms and clarifying professional roles in sustainability decisions may also be essential. Overall, mapping our findings to the TDF suggests that effective strategies will require environmental and contextual changes to support a responsible transition away from disposables.

Together, these findings may enable translation of identified enablers and barriers into strategies to reduce dis-incentives for reusable trocar use. At the device level, design improvements addressing air leakage, fixation, and ergonomics—ideally informed by early and diverse end-user involvement—may enhance usability and acceptance. At the institutional level, engagement of hospital leadership and procurement departments is essential; embedding sustainability criteria into purchasing decisions, integrating reusable trocars into standard instrument trays, and aligning with CSSD capacity can reduce workflow disruption and normalize use. Training and exposure should be strengthened through early incorporation into residency programs, hands-on training, and senior surgeon role modelling. At the cultural level, shifting defaults—such as limiting routine availability of disposable trocars—may be more effective than voluntary adoption alone. Finally, locally implemented sustainability policies may support more consistent use.

This study included surgeons and residents from diverse hospital types, applied a validated behavioral framework, and achieved thematic saturation. Conducted within the Dutch healthcare system, findings may not generalize to countries with different sterilization logistics, procurement practices, surgical culture or hospital norms. Only end-users were included, excluding organizational stakeholders such as procurement officers and CSSD staff; therefore, findings reflect clinicians’ perceptions of institutional and logistical processes and may simplify organizational constraints and decision-making dynamics. Conclusions related to system-level barriers should thus be interpreted with caution, and future research should include these stakeholders. Some participants were aware of the interviewer’s sustainability research, which may have introduced social desirability bias despite mitigation strategies. Additionally, convenience and snowball sampling may have enriched the sample with sustainability-interested clinicians, potentially overrepresenting positive perspectives toward reusable trocars, and findings should be interpreted accordingly. However, given the exploratory, theory-driven aim of this qualitative study, the objective was to identify relevant enablers and barriers in depth rather than to estimate prevalence. Findings were limited to general surgery; other specialties may present unique barriers.

This study identified key enablers and barriers to the use of reusable trocars. Challenges—such as usability, workflow disruptions, and limited clinician involvement in decision-making—appeared to stem more from environmental and contextual factors, including tray setup, training, and procurement policies, than from the devices themselves. Using the TDF, we demonstrated that trocar use is shaped primarily by the domains Beliefs about Consequences, Environmental Context and Resources, Skills, and Social/Professional Role and Identity. Participants emphasized that success implementation will require coordinated, multi-level strategies. When embedded effectively, reusable trocars may serve as a practical, scalable, high-impact intervention to reduce surgical emissions.

## Supplementary Information

Below is the link to the electronic supplementary material.

Supplementary file1 (DOCX 30 KB)

Supplementary file2 (DOCX 28 KB)

Supplementary file3 (DOCX 16 KB)

Supplementary file4 (XLSX 23 KB)

## References

[1] Romanello M di Napoli C, Green C, Kennard H, Lampard P, Scamman D, Walawender M, Ali Z, Ameli N, Ayeb-Karlsson S et al (2023) The 2023 report of the Lancet Countdown on health and climate change: the imperative for a health-centred response in a world facing irreversible harms. Lancet 402(10419):2346–2394. 10.1016/S0140-6736(23)01859-7 10.1016/S0140-6736(23)01859-7 PMC7616810 37977174

[2] Romanello M, McGushin A, Di Napoli C, Drummond P, Hughes N, Jamart L, Kennard H, Lampard P, Solano Rodriguez B, Arnell N et al (2021) The 2021 report of the Lancet Countdown on health and climate change: code red for a healthy future. Lancet 398(10311):1619–1662. 10.1016/S0140-6736(21)01787-6 34687662 10.1016/S0140-6736(21)01787-6 PMC7616807

[3] Watts N, Amann M, Arnell N, Ayeb-Karlsson S, Belesova K, Berry H, Bouley T, Boykoff M, Byass P, Cai W et al (2018) The 2018 report of the Lancet Countdown on health and climate change: shaping the health of nations for centuries to come. Lancet 392(10163):2479–2514. 10.1016/S0140-6736(18)32594-7 30503045 10.1016/S0140-6736(18)32594-7 PMC7616804

[4] Pichler P-P, Jaccard I, Weisz U, Weisz H (2019) International comparison of health care carbon footprints. Environ Res Lett. 10.1088/1748-9326/ab19e1

[5] Steenmeijer MA, Rodrigues JFD, Zijp MC, van der Waaijers- Loop SL (2022) The environmental impact of the Dutch health-care sector beyond climate change: an input–output analysis. Lancet Planet Health 6(12):e949–e957. 10.1016/S2542-5196(22)00244-3. Accessed 8 Jan 2025 36495889 10.1016/S2542-5196(22)00244-3

[6] Lenzen M, Malik A, Li M, Fry J, Weisz H, Pichler PP, Chaves LSM, Capon A, Pencheon D (2020) The environmental footprint of health care: a global assessment. Lancet Planet Health 4(7):e271–e279. 10.1016/s2542-5196(20)30121-2 32681898 10.1016/S2542-5196(20)30121-2

[7] Eckelman MJ, Sherman J (2016) Environmental impacts of the U.S. health care system and effects on public health. PLoS ONE 11(6):e0157014. 10.1371/journal.pone.0157014 27280706 10.1371/journal.pone.0157014 PMC4900601

[8] Thiel CL, Eckelman M, Guido R, Huddleston M, Landis AE, Sherman J, Shrake SO, Copley-Woods N, Bilec MM (2015) Environmental impacts of surgical procedures: life cycle assessment of hysterectomy in the United States. Environ Sci Technol 49(3):1779–1786. 10.1021/es504719g 25517602 10.1021/es504719g PMC4319686

[9] Lee B-K, Ellenbecker MJ, Moure-Eraso R (2002) Analyses of the recycling potential of medical plastic wastes. Waste Manag 22(5):461–470. 10.1016/S0956-053X(02)00006-5 12092754 10.1016/s0956-053x(02)00006-5

[10] Penn E, Yasso SF, Wei JL (2012) Reducing disposable equipment waste for tonsillectomy and adenotonsillectomy cases. Otolaryngol Head Neck Surg 147(4):615–618. 10.1177/0194599812450681 22675005 10.1177/0194599812450681

[11] Taylor AS, Au S, Krivankova B, Asanai K, Manimaran N (2023) Carbon footprint of laparoscopic right hemicolectomy. Br J Surg. 10.1093/bjs/znad422. Accessed 14 Nov 2024 38123466 10.1093/bjs/znad422

[12] Chan KS, Lo HY, Shelat VG (2023) Carbon footprints in minimally invasive surgery: good patient outcomes, but costly for the environment. World J Gastrointest Surg 15(7):1277–1285. 10.4240/wjgs.v15.i7.1277 37555111 10.4240/wjgs.v15.i7.1277 PMC10405111

[13] Rizan C, Steinbach I, Nicholson R, Lillywhite R, Reed M, Bhutta MF (2020) The carbon footprint of surgical operations: a systematic review. Ann Surg 272(6):986–995. 10.1097/sla.0000000000003951 32516230 10.1097/SLA.0000000000003951

[14] MacNeill AJ, Lillywhite R, Brown CJ (2017) The impact of surgery on global climate: a carbon footprinting study of operating theatres in three health systems. Lancet Planet Health 1(9):e381–e388. 10.1016/S2542-5196(17)30162-6 29851650 10.1016/S2542-5196(17)30162-6

[15] Latta M, Shaw C, Gale J (2021) The carbon footprint of cataract surgery in Wellington. N Z Med J 134(1541):13–21 34531593

[16] Grinberg D, Buzzi R, Pozzi M, Schweizer R, Capsal JF, Thinot B, Quyen Le M, Obadia JF, Cottinet PJ (2021) Eco-audit of conventional heart surgery procedures. Eur J Cardiothorac Surg 60(6):1325–1331. 10.1093/ejcts/ezab320 34411226 10.1093/ejcts/ezab320

[17] Ferrero A, Thouvenin R, Hoogewoud F, Marcireau I, Offret O, Louison P, Monnet D, Brézin AP (2022) The carbon footprint of cataract surgery in a French University Hospital. J Fr Ophtalmol 45(1):57–64. 10.1016/j.jfo.2021.08.004 34823888 10.1016/j.jfo.2021.08.004

[18] Hubert J, Gonzalez-Ciccarelli LF, Wang AW, Toledo E, Ferrufino R, Smalls K, Brovman E, Cobey FC (2022) Carbon emissions during elective coronary artery bypass surgery, a single center experience. J Clin Anesth 80:110850. 10.1016/j.jclinane.2022.110850 35525051 10.1016/j.jclinane.2022.110850

[19] Ditac G, Cottinet PJ, Quyen Le M, Grinberg D, Duchateau J, Gardey K, Dulac A, Delinière A, Haddad C, Boussuge-Roze J et al (2023) Carbon footprint of atrial fibrillation catheter ablation. Europace 25(2):331–340. 10.1093/europace/euac160 36107465 10.1093/europace/euac160 PMC10103577

[20] Robinson PN, Surendran K, Lim SJ, Robinson M (2023) The carbon footprint of surgical operations: a systematic review update. Ann R Coll Surg Engl 105(8):692–708. 10.1308/rcsann.2023.0057 37906978 10.1308/rcsann.2023.0057 PMC10626532

[21] Rizan C, Bhutta MF (2022) Environmental impact and life cycle financial cost of hybrid (reusable/single-use) instruments versus single-use equivalents in laparoscopic cholecystectomy. Surg Endosc 36(6):4067–4078. 10.1007/s00464-021-08728-z 34559257 10.1007/s00464-021-08728-z PMC9085686

[22] Eussen MMM, Bluiminck S, Chambon M, Batcup C, Kooistra EJ, Stobernack T, van Harreveld F, Bouvy ND, de Reuver PR (2025) Perspectives on disposable and reusable surgical materials in laparoscopic surgery: a global survey amongst surgeons. Int J Surg. 10.1097/js9.0000000000002442 40358663 10.1097/JS9.0000000000002442

[23] Comes DJ, Bluiminck S, Kooistra EJ, de Nes L, van Workum F, Touw H, Eussen MMM, Bouvy ND, Stobernack T, de Reuver PR (2024) The carbon footprint of a laparoscopic cholecystectomy. Br J Surg. 10.1093/bjs/znae225 39291674 10.1093/bjs/znae225 PMC11408924

[24] Boberg L, Singh J, Montgomery A, Bentzer P (2022) Environmental impact of single-use, reusable, and mixed trocar systems used for laparoscopic cholecystectomies. PLoS ONE 17(7):e0271601. 10.1371/journal.pone.0271601 35839237 10.1371/journal.pone.0271601 PMC9286249

[25] Lim BLS, Narayanan V, Nah SA (2023) Knowledge, attitude, and practices of operating theatre staff towards environmentally sustainable practices in the operating theatres. Pediatr Surg Int 39(1):152. 10.1007/s00383-023-05400-6 36930355 10.1007/s00383-023-05400-6

[26] Polivka BJ, Chaudry RV, Mac Crawford J (2012) Public health nurses’ knowledge and attitudes regarding climate change. Environ Health Perspect 120(3):321–325. 10.1289/ehp.1104025 22128069 10.1289/ehp.1104025 PMC3295355

[27] Kotcher J, Maibach E, Miller J, Campbell E, Alqodmani L, Maiero M, Wyns A (2021) Views of health professionals on climate change and health: a multinational survey study. Lancet Planet Health 5(5):e316–e323. 10.1016/s2542-5196(21)00053-x 33838130 10.1016/S2542-5196(21)00053-X PMC8099728

[28] Kalogirou MR, Dahlke S, Davidson S, Yamamoto S (2020) Nurses’ perspectives on climate change, health and nursing practice. J Clin Nurs 29(23–24):4759–4768. 10.1111/jocn.15519 33010079 10.1111/jocn.15519

[29] López-Medina IM, Álvarez-García C, Parra-Anguita L, Sanz-Martos S, Álvarez-Nieto C (2022) Perceptions and concerns about sustainable healthcare of nursing students trained in sustainability and health: a cohort study. Nurse Educ Pract 65:103489. 10.1016/j.nepr.2022.103489 36343526 10.1016/j.nepr.2022.103489

[30] Dal Mas F, Cobianchi L, Piccolo D, Balch J, Biancuzzi H, Biffl WL, Campostrini S, Cicuttin E, Coccolini F, Damaskos D et al (2024) Are we ready for “green surgery” to promote environmental sustainability in the operating room? Results from the WSES STAR investigation. World J Emerg Surg 19(1):5. 10.1186/s13017-024-00533-y 38267949 10.1186/s13017-024-00533-y PMC10809586

[31] Sathe TS, Alseidi A, Bellato V, Ganjouei AA, Foroutani L, Hall RP, Potapov O, Bello RJ, Johnson SM, Marconi S et al (2024) Perspectives on sustainability among surgeons: findings from the SAGES-EAES sustainability in surgical practice task force survey. Surg Endosc. 10.1007/s00464-024-11137-7 39160314 10.1007/s00464-024-11137-7 PMC11458713

[32] Harris H, Bhutta MF, Rizan C (2021) A survey of UK and Irish surgeons’ attitudes, behaviours and barriers to change for environmental sustainability. Ann R Coll Surg Engl 103(10):725–729. 10.1308/rcsann.2021.0271 34719956 10.1308/rcsann.2021.0271 PMC10335270

[33] Plesk P (2021) Appendix B: Redesigning health care with insights from the science of complex adaptive systems. National Academy of Sciences

[34] Lam K, Gadi N, Acharya A, Winter Beatty J, Darzi A, Purkayastha S (2023) Interventions for sustainable surgery: a systematic review. Int J Surg 109(5):1447–1458. 10.1097/js9.0000000000000359 37042311 10.1097/JS9.0000000000000359 PMC10389594

[35] Ledda V, George C, Glasbey J, Labib P, Li E, Lu A, Kudrna L, Nepogodiev D, Picciochi M, Williams I, Bhangu A (2024) Uncertainties and opportunities in delivering environmentally sustainable surgery: the surgeons’ view. Anaesthesia 79(3):293–300. 10.1111/anae.16195 38207004 10.1111/anae.16195

[36] Almukhtar A, Batcup C, Bowman M, Winter-Beatty J, Leff D, Demirel P, Porat T, Judah G (2024) Barriers and facilitators to sustainable operating theatres: a systematic review using the Theoretical Domains Framework. Int J Surg 110(1):554–568. 10.1097/js9.0000000000000829 37889570 10.1097/JS9.0000000000000829 PMC10793789

[37] Batcup C, Breth-Petersen M, Dakin T, Barratt A, McGain F, Newell BR, Pickles K (2023) Behavioural change interventions encouraging clinicians to reduce carbon emissions in clinical activity: a systematic review. BMC Health Serv Res 23(1):384. 10.1186/s12913-023-09370-2 37081553 10.1186/s12913-023-09370-2 PMC10116654

[38] Frick M, Neu L, Liebhaber N, Sperner-Unterweger B, Stötter J, Keller L, Hüfner K (2021) Why do we harm the environment or our personal health despite better knowledge? The knowledge action gap in healthy and climate-friendly behavior. Sustainability 13(23):13361

[39] Steg L, Bolderdijk JW, Keizer K, Perlaviciute G (2014) An integrated framework for encouraging pro-environmental behaviour: the role of values, situational factors and goals. J Environ Psychol 38:104–115. 10.1016/j.jenvp.2014.01.002

[40] Mazer L, Sandhu G (2021) The case for Social Science Research in Surgery. JAMA Surg 156(5):411–412. 10.1001/jamasurg.2020.6671. Accessed 6 Nov 2025 33566080 10.1001/jamasurg.2020.6671

[41] Boyer CJ, Bowen K, Murray V, Hadley J, Hilly JJ, Hess JJ, Ebi KL (2020) Using implementation science for health adaptation: opportunities for Pacific Island Countries. Health Aff (Millwood) 39(12):2160–2167. 10.1377/hlthaff.2020.01101 33284708 10.1377/hlthaff.2020.01101

[42] Neta G, Pan W, Ebi K, Buss DF, Castranio T, Lowe R, Ryan SJ, Stewart-Ibarra AM, Hapairai LK, Sehgal M et al (2022) Advancing climate change health adaptation through implementation science. Lancet Planet Health 6(11):e909–e918. 10.1016/s2542-5196(22)00199-1 36370729 10.1016/S2542-5196(22)00199-1 PMC9669460

[43] Cane J, O’Connor D, Michie S (2012) Validation of the theoretical domains framework for use in behaviour change and implementation research. Implement Sci 7(1):37. 10.1186/1748-5908-7-37 22530986 10.1186/1748-5908-7-37 PMC3483008

[44] Davies JF, McGain F, Sloan E, Francis J, Best S (2024) A qualitative exploration of barriers, enablers, and implementation strategies to replace disposable medical devices with reusable alternatives. Lancet Planet Health 8(11):e937–e945. 10.1016/s2542-5196(24)00241-9 39515352 10.1016/S2542-5196(24)00241-9

[45] Chambon M, Eussen MMM, Bluminck S, Batcup C, Dalege J, de Wit S, Bouvy ND, de Reuver PR, van Harreveld F (in press) Promoting environmentally sustainable behavior in healthcare: changing specific behavior requires measuring specific behavior. Cross-sectional study. Int Surg J

[46] Malterud K, Siersma VD, Guassora AD (2016) Sample size in qualitative interview studies: guided by information power. Qual Health Res 26(13):1753–1760. 10.1177/1049732315617444 26613970 10.1177/1049732315617444

[47] Francis JJ, Johnston M, Robertson C, Glidewell L, Entwistle V, Eccles MP, Grimshaw JM (2010) What is an adequate sample size? Operationalising data saturation for theory-based interview studies. Psychol Health 25(10):1229–1245. 10.1080/08870440903194015 20204937 10.1080/08870440903194015

[48] Vasileiou K, Barnett J, Thorpe S, Young T (2018) Characterising and justifying sample size sufficiency in interview-based studies: systematic analysis of qualitative health research over a 15-year period. BMC Med Res Methodol 18(1):148. 10.1186/s12874-018-0594-7 30463515 10.1186/s12874-018-0594-7 PMC6249736

[49] Tong A, Sainsbury P, Craig J (2007) Consolidated criteria for reporting qualitative research (COREQ): a 32-item checklist for interviews and focus groups. Int J Qual Health Care 19(6):349–357. 10.1093/intqhc/mzm042. Accessed 27 Aug 2025 17872937 10.1093/intqhc/mzm042

[50] Atkins L, Francis J, Islam R, O’Connor D, Patey A, Ivers N, Foy R, Duncan EM, Colquhoun H, Grimshaw JM et al (2017) A guide to using the Theoretical Domains Framework of behaviour change to investigate implementation problems. Implement Sci 12(1):77. 10.1186/s13012-017-0605-9 28637486 10.1186/s13012-017-0605-9 PMC5480145

[51] O’Connor C, Joffe H (2020) Intercoder reliability in qualitative research: debates and practical guidelines. Int J Qual Methods 19:1609406919899220. 10.1177/1609406919899220

[52] Kloevekorn L, Roemeling O, Fakha A, Hage E, Smailhodzic E (2024) Decarbonizing surgical care: a qualitative systematic review guided by the Congruence Model. BMC Health Serv Res 24(1):1456. 10.1186/s12913-024-11929-6 39580403 10.1186/s12913-024-11929-6 PMC11585939

[53] Money AG, Barnett J, Kuljis J, Craven MP, Martin JL, Young T (2011) The role of the user within the medical device design and development process: medical device manufacturers’ perspectives. BMC Med Inform Decis Mak 11(1):15. 10.1186/1472-6947-11-15 21356097 10.1186/1472-6947-11-15 PMC3058010

[54] Aboueid S, Beyene M, Nur T (2023) Barriers and enablers to implementing environmentally sustainable practices in healthcare: a scoping review and proposed roadmap. Healthc Manage Forum 36(6):405–413. 10.1177/08404704231183601 37357691 10.1177/08404704231183601 PMC10604425

[55] Zurynski Y, Ludlow K, Testa L, Augustsson H, Herkes-Deane J, Hutchinson K, Lamprell G, McPherson E, Carrigan A, Ellis LA et al (2023) Built to last? Barriers and facilitators of healthcare program sustainability: a systematic integrative review. Implement Sci 18(1):62. 10.1186/s13012-023-01315-x 37957669 10.1186/s13012-023-01315-x PMC10641997

[56] Schwab R, Schiestl LJ, Hasenburg A (2025) Greening the future of healthcare: implementation of sustainability strategies in German hospitals and beyond-a review. Front Public Health 13:1559132. 10.3389/fpubh.2025.1559132 40385621 10.3389/fpubh.2025.1559132 PMC12082835

[57] HealthManagement.org. Advancing Sustainability in Healthcare Procurement. 2025.

[58] Kaiser Permanente (2024) Catalyzing collection action to decarbonize healthcare

[59] Lavoie DCT et al (2024) Healthcare procurement in the race to net-zero: practical steps for healthcare leadership. Health Manage Forum 37(5):384–389 10.1177/08404704241258152 PMC11348617 39033434

[60] Cramer J (2022) Building a circular future. A publication of the Amsterdam Economic Board in cooperation with Holland Circular Hotspot. https://circulareconomy.europa.eu/platform/sites/default/files/building-a-circular-future-jacqueline-cramer-amsterdam-economic-board.pdf. Accessed 2024

[61] Reike D, Vermeulen WJV, Witjes S (2018) The circular economy: new or refurbished as CE 3.0? — exploring controversies in the conceptualization of the circular economy through a focus on history and resource value retention options. Resour Conserv Recycl 135:246–264. 10.1016/j.resconrec.2017.08.027

[62] Nilsen P, Seing I, Ericsson C, Birken SA, Schildmeijer K (2020) Characteristics of successful changes in health care organizations: an interview study with physicians, registered nurses and assistant nurses. BMC Health Serv Res 20(1):147. 10.1186/s12913-020-4999-8 32106847 10.1186/s12913-020-4999-8 PMC7045403

[63] Moustafa Saleh MS, Elsabahy HE, Abdel-Sattar SA-L, Abd-Elhamid ZN, Al Thobaity A, Mohammed Aly SM, Shokry WM (2024) Fostering green transformational leadership: the influence of green educational intervention on nurse managers’ green behavior and creativity. BMC Nurs 23(1):393. 10.1186/s12912-024-01991-0 38849843 10.1186/s12912-024-01991-0 PMC11157831

[64] Liao Y (2022) Sustainable leadership: a literature review and prospects for future research. Front Psychol 13:1045570. 10.3389/fpsyg.2022.1045570 36420377 10.3389/fpsyg.2022.1045570 PMC9676637

[65] Jones NL, Gilman SE, Cheng TL, Drury SS, Hill CV, Geronimus AT (2019) Life course approaches to the causes of health disparities. Am J Public Health 109(S1):S48-s55. 10.2105/ajph.2018.304738 30699022 10.2105/AJPH.2018.304738 PMC6356123

[66] Cohen ES, Kringos DS, Grandiek F, Kouwenberg L, Sperna Weiland NH, Richie C, Hehenkamp WJK, Aarts JWM (2025) Patients’ attitudes towards integrating environmental sustainability into healthcare decision‐making: an interview study. Health Expect 28(1):e70155. 10.1111/hex.70155 39828934 10.1111/hex.70155 PMC11743189

[67] Resnik DB, Pugh J (2024) Green bioethics, patient autonomy and informed consent in healthcare. J Med Ethics 50(7):489–493. 10.1136/jme-2023-109404 37833040 10.1136/jme-2023-109404 PMC11014890

[68] Cohen ES, Kringos DS, Hehenkamp WJK, Richie C (2024) Harmonising green informed consent with autonomous clinical decision-making: a reply to Resnik and Pugh. J Med Ethics 50(7):498–500. 10.1136/jme-2024-109863 38290854 10.1136/jme-2024-109863

