# Supplementary material for: Enablers and barriers for using reusable trocars: a qualitative study of surgeons’ and residents’ perspectives
Source: Surg Endosc. 2026 Jan 26;40(4):3106–23. doi: 10.1007/s00464-026-12571-5 (PMC13053580; doi:10.1007/s00464-026-12571-5)
Supplement: Supplementary file 1 — Supplementary file1 (DOCX 30 KB) [file 464_2026_12571_MOESM1_ESM.docx]

# Appendix A: Semi-structured interview guide

This guide presents the English translation of the original interview protocol, which was developed and conducted in Dutch.

## Introduction

Thanking the participant and introducing yourself:

- Thank you for taking the time to participate in this study.
- My name is [name], and I am a [role] at [organization]. I will be conducting this interview today.

Provide a brief overview of the study:

- This research is part of the CAREFREE consortium, which focuses on making healthcare more sustainable. One of the topics we are studying is the use of medical instruments in surgery.
- In this interview, we aim to explore what surgeons and residents think about different types of laparoscopic trocars.

Explain the structure of the interview and confirm language preference:

- We’ll start by explaining the informed consent procedure and then begin the recording. After that, we’ll ask a few background questions, followed by the main part of the interview.
- May I address you informally as ‘you’ during our conversation?

Informed consent and recording procedure:

- You received the informed consent form by email prior to this interview. Were you able to review it, and do you agree with its contents?
- If so, I will now start the recording and repeat the consent question to ensure your verbal agreement is documented. After that, we will begin the interview.

*— Start recording —*

Obtain verbal consent:

- You previously received the informed consent form. Do you confirm that you have read and agreed to its contents, and that you consent to this interview being recorded?
- Thank you. We will now begin the interview.

Provide interview instructions:

- Please try to give brief but complete answers.
- The questions will focus on laparoscopic trocars.

## Background questions

We’ll begin with a few personal questions to understand your background as a participant in this study.

- What is your current position, are you a surgeon or a resident?
- At which hospital(s) do you currently work?
- How many years have you been in your current role? Please round up to the nearest year.
- How many years have you been performing laparoscopic surgeries? Please round up to the nearest year.
- Would you be willing to share your age?
- How would you describe your gender?

## Current use

The following questions explore your experience with disposable and reusable laparoscopic trocars.

- Do you have experience with reusable trocars?
  - If yes: How much experience do you have (e.g., how many years)?
  - If no: Please answer the following questions based on your expectations of reusable trocars.
- Do you prefer disposable or reusable trocars? Why?
- What is the policy in your hospital regarding the use of disposable and reusable trocars? Are both types available? Are both permitted for use?
- Can you personally choose between disposable and reusable trocars?
  - If not:
    - Why not?
    - Is there a standard or default choice? If so, whose choice is it, yours or someone else’s?
  - If yes:
    - Do you alternate between them? Approximately how often do you use each type? Can you estimate the percentage of cases in which you use disposable vs. reusable trocars?
    - What factors influence your choice? Does the type of procedure play a role?
    - To what extent do you consciously consider this choice?
    - Does the environmental impact influence your decision? If so, how?
- Do you think other surgeons and residents in your hospital prefer disposable or reusable trocars? Why?
- If choice is allowed, how often do you think other surgeons and residents in your hospital would choose each type? Do you know their standard preference?
  - Do you think these patterns are representative of other hospitals in the Netherlands? Why or why not?
  - Have you worked in other hospitals where different practices were followed?
- How important do you think it is to replace disposable trocars with reusable ones?
- What barriers do you foresee in a full transition to reusable trocars?
  - For yourself?
  - For other surgeons and/or residents?
  - For other stakeholders?
- What factors could facilitate such a transition?
  - For yourself?
  - For other surgeons and/or residents?
  - For other stakeholders?
- If you wanted to use reusable trocars more often, would that be possible?
  - Do you have the autonomy to make that decision? If not, why not?
  - Do you have the necessary resources? If not, why not?
- Do you think your colleagues experience social pressure to use reusable trocars more often?
- In your view, how much more effort is required to clean reusable trocars compared to disposable ones?

## Beliefs

The following questions explore your views on different types of laparoscopic trocars. If relevant, you may distinguish between 5 mm and 10-12 mm trocars.

- Disposable trocars
  - What do you consider to be the advantages of using disposable trocars?
  - What do you consider to be the disadvantages?
- Reusable trocars
  - What do you consider to be the advantages of using reusable trocars?
  - What do you consider to be the disadvantages?
- How much difference do you think switching from disposable to reusable trocars would make in reducing environmental impact?
- Are you already familiar with the updated reusable trocars from B. Braun from the CAREFREE project?
  - If so, how? What do you know about them?
  - What do you expect the advantages of the updated reusable trocars to be?
  - What do you expect the disadvantages to be?
  - Do you feel confident in your ability to use the updated reusable trocars?
- If the respondent has not mentioned trocar sizes spontaneously: Are there important differences in the advantages or disadvantages between 5 mm and 10-12 mm trocars?

## Expectations regarding the updated reusable trocars

The following questions explore your expectations about the updated reusable trocars that you will be using in the CAREFREE project.

- Do you think you would like to continue using the updated reusable trocars after the clinical use phase? Why or why not?
- Do you expect that other surgeons and residents in your hospital would want to continue using them? Why or why not?
- Do you think it will be feasible to continue using these trocars after the clinical phase? Why or why not?
- To what extent does the brand or manufacturer of the trocar influence your expectations or perceptions of the instrument?
- What is your opinion on the clinical use phase in which the updated reusable trocars are mandated for use?

## Emotions

These questions explore emotional responses related to switching to the updated reusable trocars.

Note for the interviewer: These questions aim to uncover emotions such as enthusiasm, resistance, or concern. If the respondent does not spontaneously mention feelings, consider prompting with examples, only if needed.

- In your view, how might people feel about switching to the updated reusable trocars?
  - What about other surgeons and residents?
  - What about other stakeholders such as procurement or sterilization departments?
  - What about patients?
    - How do you personally feel about the idea of switching to the updated reusable trocars?
    - Do you have any concerns about using the updated reusable trocars? If yes, what are they?

## Decision-making around transition to reusable trocars

- In your opinion, who is currently responsible for the final decision about transitioning to reusable trocars?
- Who do you think should be make the final decision for transitioning to reusable trocars?
- Who do you think should be involved in making that decision for transitioning to reusable trocars?
- What do you think are the most important factors to consider when deciding whether to switch to reusable trocars?

## Automatism

These questions explore how automated trocar use is in your daily practice and how switching to updated instruments might impact that.

- To what extent is the use of laparoscopic trocars automatic for you in your current practice?
  - How much do you need to think about using trocars during surgery?
  - How much effort does using trocars typically require?
  - Does the use of disposable and reusable trocars feel different in terms of how automatic it is for you?
- How much effort do you think it will take to switch to the updated reusable trocars? Why?
  - What aspects of that process do you think will be the most demanding?
- How do you feel about having to adjust your working methods due to decisions made by others (e.g., hospital management), such as mandatory use of reusable trocars?

## Knowledge

We’ll conclude with a few questions about the environmental impact of healthcare.

- Are you familiar with the environmental impact of healthcare, particularly in the operating room and related to the use of disposable products?
- Could you describe what you already know about this topic?

## Closing

- - - Is there anything we haven’t covered that you’d like to add?
    - Thank you very much for your time and insights.

*— End recording —*
