# Supplementary material for: Enablers and barriers for using reusable trocars: a qualitative study of surgeons’ and residents’ perspectives
Source: Surg Endosc. 2026 Jan 26;40(4):3106–23. doi: 10.1007/s00464-026-12571-5 (PMC13053580; doi:10.1007/s00464-026-12571-5)
Supplement: Supplementary file 2 — Supplementary file2 (DOCX 28 KB) [file 464_2026_12571_MOESM2_ESM.docx]

# Appendix B: Theoretical Domains Framework (TDF) codebook

The TDF codebook is based on the definitions and constructs outlined by Atkins et al. (2017) and has been adapted by researcher ME and MK for application in the proposed interviews.

| **Label** | **Definition & *example*** | **Constructs** |
| --- | --- | --- |
| ***Knowledge*** | An awareness of the existence of something  *Knowledge of reusable trocars. Knowledge of the environmental impact of the healthcare, operating room and disposable trocars.* | Knowledge (including knowledge of condition/scientific rationale)  Procedural knowledge  Knowledge of task environment |
| ***Skills*** | An ability or proficiency acquired through practice  *The competence to handle reusable trocars during surgery. The effort required to develop the skills needed to transition from disposable to reusable trocars.* | Skills Skills development  Competence ability  Interpersonal skills  Practice  Skill assessment |
| ***Social/professional role and identity*** | A coherent set of behaviours and displayed personal qualities of an individual in a social or work setting  *The role and responsibilities of surgeons in the procurement and use of disposable and reusable trocars. Identifying other stakeholders and their roles in decisions regarding the procurement and use of disposable and reusable trocars. The role of leadership in facilitating the transition from disposable to reusable trocars.* | Professional identity  Professional role  Social identity  Identity  Professional boundaries  Professional confidence  Group identity  Leadership  Organisational commitment |
| ***Beliefs about capabilities*** | Acceptance of the truth, reality or validity about an ability, talent or facility that a person can put to constructive use  *Belief that surgeons could use reusable trocars more often if desired. Belief that surgeons don’t need new skills to use reusable trocars effectively. Belief that reusable trocars won’t disrupt the automatic routine of trocar insertion. Belief that surgeons will continue using reusable trocars after a test period.* | Self-confidence  Perceived competence self-efficacy  Perceived behavioural control  Beliefs  Self-esteem  Empowerment  Professional confidence |
| ***Optimism*** | The confidence that things will happen for the best or that desired goals will be attained  *Confidence that reusable trocars will replace disposable ones. Pessimism that reusable trocars won’t be preferred over disposables.* | Optimism  Pessimism  Unrealistic optimism  Identity |
| ***Beliefs about consequences*** | Acceptance of the truth, reality, or validity about outcomes of a behaviour in a given situation  *Beliefs about the cost-effectiveness and environmental sustainability of reusable trocars. Beliefs about patient safety, efficiency, or equipment failures when using reusable trocars instead of disposables. Beliefs about disposable equipment, including supply chain disruptions, storage, and sterility. Beliefs about waste from disposable equipment and its packaging.* | Beliefs  Outcome expectancies  Characteristics of outcome expectancies  Anticipated regret  Consequents |
| ***Reinforcement*** | Increasing the probability of a response by arranging a dependent relationship, or contingency, between the response and a given stimulus  *Incentives that influence whether a decision-maker chooses to procure reusable trocars instead of disposables.* | Rewards  Incentives  Punishment  Consequents  Reinforcement  Contingencies  Sanctions |
| ***Intentions*** | A conscious decision to perform a behaviour or a resolve to act in a certain way  *Determination to successfully introduce reusable trocars. Determination to continue using them. Intentions to change procurement, supply, or usage. Staff actively choosing reusable trocars.* | Stability of intentions  Stages of change model  Transtheoretical model and stages of change |
| ***Goals*** | Mental representations of outcomes or end states that an individual wants to achieve  *Prioritization of reusable trocars. Identifying steps toward increasing adoption. Prioritizing sustainability in decision-making or failing to do so.* | Goals (distal/proximal)  Goal priority  Goal/target setting  Goals (autonomous/controlled)  Action planning  Implementation intention |
| ***Memory, attention and decision processes*** | The ability to retain information, focus selectively on aspects of the environment and choose between two or more alternatives  *Explains the decision-making process (if applicable to their role) in choosing between disposable and reusable options. Describes habitual behaviors and routines regarding the use of trocars.* | Memory  Attention  Attention control  Decision making  Cognitive overload/tiredness |
| ***Environmental context and resources*** | Any circumstance of a person’s situation or environment that discourages or encourages the development of skills and abilities, independence, social competence and adaptive behaviour  *Describes operational factors within operating theatres, including local hospital policies, sterilization capacity, cost, manufacturer type, and other perceived barriers.* | Environmental stressors  Resources/material resources  Organisational culture/climate  Salient events/critical incidents  Person × environment interaction  Barriers and facilitators |
| ***Social influences*** | Those interpersonal processes that can cause individuals to change their thoughts, feelings, or behaviours  *Describes perceptions of colleagues’ willingness to switch to reusable trocars and the influence of decision-makers in approving their use. Explores social influences on adopting reusable trocars, including waste reduction activities, motivations for sustainability, and the impact of peer influence. Also considers the role of behavior modeling in encouraging sustainable practices.* | Social pressure  Social norms  Group conformity  Social comparisons  Group norms  Social support  Power  Intergroup conflict  Alienation  Group identity  Modelling |
| ***Emotion*** | A complex reaction pattern, involving experiential, behavioural, and physiological elements, by which the individual attempts to deal with a personally significant matter or event  *Describes both excitement and anxiety regarding the increased use of reusable trocars. Explores stress related to their implementation, e.g. the additional sterilization workload.* | Fear  Anxiety  Affect  Stress  Depression  Positive/negative affect  Burn-out |
| ***Behavioural regulation*** | Anything aimed at managing or changing objectively observed or measured actions  *Describes the circumstances under which reusable trocars or disposable alternatives are chosen. Explores the willingness or resistance to changing established procedures and habits, as well as the processes that influence routine behavior.* | Self-monitoring  Breaking habit  Action planning |
