# Supplementary material for: Enablers and barriers for using reusable trocars: a qualitative study of surgeons’ and residents’ perspectives
Source: Surg Endosc. 2026 Jan 26;40(4):3106–23. doi: 10.1007/s00464-026-12571-5 (PMC13053580; doi:10.1007/s00464-026-12571-5)
Supplement: Supplementary file 3 — Supplementary file3 (DOCX 16 KB) [file 464_2026_12571_MOESM3_ESM.docx]

**Appendix C: Participant Characteristics**

|  | **n** | **%** |
| --- | --- | --- |
| *Gender* | | |
| Male | 14 | 70% |
| Female | 6 | 30% |
| *Current function* | | |
| Resident | 8 | 40% |
| Attending | 12 | 60% |
| *Hospital type* | | |
| Academic | 10 | 50% |
| Large general | 8 | 40% |
| Small general | 2 | 10% |
|  | **Median** | **IQR** |
| Age (years) | 37.00 | 12 |
| Experience in current role (years) | 5.75 | 7.75 |
| Experience in laparoscopic surgery (years) | 8.00 | 14 |
| Recording time (minutes) | 26.52 | 8.83 |
